# Distribution of Long-Range Linkage Disequilibrium and Tajima’s D Values in Scandinavian Populations of Norway Spruce (*Picea abies*)

**DOI:** 10.1534/g3.112.005462

**Published:** 2013-05-01

**Authors:** Hanna Larsson, Thomas Källman, Niclas Gyllenstrand, Martin Lascoux

**Affiliations:** * Department of Ecology and Genetics, EBC, Uppsala University, 752 36 Uppsala, Sweden; † Department of Plant Biology and Forest Genetics, Swedish University of Agricultural Sciences, Uppsala, 750 07 Uppsala, Sweden

**Keywords:** linkage disequilibrium, conifer, recombination, Tajima’s D, resampling

## Abstract

The site frequency spectrum of mutations (SFS) and linkage disequilibrium (LD) are the two major sources of information in population genetics studies. In this study we focus on the levels of LD and the SFS and on the effect of sample size on summary statistics in 10 Scandinavian populations of Norway spruce. We found that previous estimates of a low level of LD were highly influenced by both sampling strategy and the fact that data from multiple loci were analyzed jointly. Estimates of LD were in fact heterogeneous across loci and increased within individual populations compared with the estimate from the total data. The variation in levels of LD among populations most likely reflects different demographic histories, although we were unable to detect population structure by using standard approaches. As in previous studies, we also found that the SFS-based test Tajima’s D was highly sensitive to sample size, revealing that care should be taken to draw strong conclusions from this test when sample size is small. In conclusion, the results from this study are in line with recent studies in other conifers that have revealed a more complex and variable pattern of LD than earlier studies suggested and with studies in trees and humans that suggest that Tajima’s D is sensitive to sample size. This has large consequences for the design of future association and population genetic studies in Norway spruce.

Population genetics inferences are primarily based on two sources of information: the site frequency spectrum of mutations (SFS) and the statistical association among those, that is, linkage disequilibrium (LD). The SFS has been the main source of information for demographic inferences and tests of selection, whereas LD is the key parameter in association mapping studies, impacting their feasibility, cost, and resolution. In the presence of long-range LD, fewer markers are required for mapping purposes, but mapping precision will be low. Conversely, if LD decays quickly, a large number of markers will be needed to cover the genome leading to a high cost but also to a high precision. Both the SFS and LD are influenced by the joint effect of biological factors, such as the rates of recombination and mutation, the demographic history of the population, and natural selection. They are therefore both highly dependent on which species, which part of the range within that species, and which parts of the genome are considered. In the present study we will focus on LD and the SFS estimated in Scandinavian populations of Norway spruce (*Picea abies*).

In general, conifers are expected to have a low level of LD or, conversely, a high population recombination rate, because their representatives are mostly outcrossing species with large effective population sizes (*e.g.*, Chen *et al.* 2010). Consistent with this notion, several multilocus studies have found LD decaying rapidly with distance between segregating sites in conifers (Brown *et al.* 2004; Krutovsky and Neale 2005; González-Martínez *et al.* 2006; Heuertz *et al.* 2006; Pyhäjärvi *et al.* 2007). This has led to the conclusion that too many markers would be required in conifers to make genome-wide association studies cost-effective (Neale and Savolainen 2004). These earlier studies were, however, based on relatively short loci (generally around 500 bp and up to 2.5 kb), and a recent review found that genome-wide recombination rates inferred from genetic mapping efforts were in fact lower in conifers than angiosperms (Jaramillo-Correa *et al.* 2010). In model plants, genome regions rich in repetitive elements have reduced levels of recombination (*e.g.*, Gaut *et al.* 2007) and the low genome-wide estimate in Jaramillo-Correa *et al.* (2010) is hypothesized to be, at least in part, the result of averaging recombination rates across conifer genomes rich in repetitive and transposable elements.

Indeed, in a recent study in the conifer *Cryptomeria japonica* the authors measured extensive LD at a distance of >100 kb in mostly noncoding DNA consisting of ~50% repetitive sequences (Moritsuka *et al.* 2012). The approach of using several loci and sampling individuals from a large distribution range to estimate a common level of LD may also have masked the variability of LD among genes that is now emerging as whole gene studies report high levels of LD in some of the genes examined (Lepoittevin *et al.* 2012; Namroud *et al.* 2010; Pavy *et al.* 2012). Notably, in *Pinus sylvestris*, in which previous studies suggested a rapid breakdown of LD, the sequencing of a group of allozyme coding loci revealed reduced recombination rates and complete linkage extending over several kilobases, possibly reflecting the presence of selection at some of those genes (Pyhäjärvi *et al.* 2011). Clearly, the genomes of conifers are less than homogenous with regards to patterns of LD, and more data on the variability of LD across species and the genome are needed to fully evaluate the appropriate method for association mapping studies.

Tajima’s D (Tajima 1989) is one of the most popular summary statistics of the SFS. It belongs to a large family of “neutrality” tests that compare different estimates of the population mutation rate, θ = 4N_e_μ (Zeng *et al.* 2006), where N_e_ is the effective population size and μ is the mutation rate. The key idea behind these tests is that some estimators of θ = 4N_e_μ will be more sensitive than others to specific departures from the standard neutral model. Comparing two estimators of θ should then indicate the type of departure experienced by the population. For example, Tajima’s D is calculated by taking the difference between the average pairwise nucleotide diversity (θ_π_), and the number of segregating sites as measured by θ_W_, the latter being more sensitive than the former to an excess of rare variants (Tajima 1989). Hence, Tajima’s D values are negative when there is an excess of rare variants and positive values are obtained when there is an excess of intermediate frequency variants. Tajima’s D, as well as other test statistics of the same family, is inherently sensitive to sample size, as θ_W_ is defined as a function of the number of segregating sites (S) and the number of individuals (n). The impact of difference in sample size on estimates of Tajima’s D is difficult to track analytically because both sampling strategy and species demographic history can have impact on the estimate (see, for example, Städler *et al.* 2009). In a study of climate-related candidate genes in six populations of Sitka spruce, Holliday *et al.* (2010) showed that estimates of Tajima’s D became more positive toward the northern leading edge of the species distribution. They suggested that sequential population bottlenecks during postglacial recolonization created this pattern with rare variants more common in the south and medium-frequency variants more common in the north.

Here, we investigate variation in estimated Tajima’s D values and the level of LD at eleven loci in 10 Scandinavian populations of Norway spruce. In a previous study of nucleotide diversity, we concluded that the species has low-to-moderate level of nucleotide diversity and a low level of LD (Heuertz *et al.* 2006). However, these data included only two loci longer than 1 kb and results were therefore mostly based on loci of short length (~500 bp). In light of the variability of LD recently revealed in other conifer species, our first aim was to examine levels of LD within longer fragments. The 10 populations used in this study are located along a north to south gradient on both sides of the Baltic Sea. Earlier studies of spruce populations from this geographical area have revealed a weak, though biologically significant, population structure, likely reflecting recolonization routes within Scandinavia following the last glacial maximum (Chen *et al.* 2012). Norway spruce recolonized Scandinavia after the last glacial maximum from Russian populations located south of the ice sheet and, possibly, from cryptic refugia in the northern part of European Russia too (Binney *et al.* 2009; Väliranta *et al.* 2011). The spread westwards followed two main recolonization routes (Tollefsrud *et al.* 2009), today reflected in a complex population genetic structure despite the generally high levels of gene flow (Heuertz *et al.* 2006; Chen *et al.* 2012).

The second aim of our study was to investigate if estimated values of Tajima’s D from different populations varied and whether the variation varied in a clinal fashion as reported from Sitka spruce. Because Tajima’s D estimates depend on sample size (Marroni *et al.* 2011; Nelson *et al.* 2012), three populations were sampled more densely and we used a resampling approach within these populations to check the reliability of Tajima’s D values obtained from small sample sizes.

## Material and Methods

The 11 genomic loci for this study were chosen from previous studies of sequence variation and gene expression in *P. abies* and the selection was based on length of locus, available primer sequences, and ease of amplification. *PaCOL1* (Heuertz *et al.* 2006), *PaMFT1*, *PaFTL1* (Karlgren *et al.* 2011), *PaPRR1*, and *PaPRR7* (Källman 2009) are hypothesized to be involved in the regulation of bud set due to sequence similarity to known genes involved in the regulation of flowering time in the model species *Arabidopsis thaliana*. The remaining loci correspond to genes exhibiting a significant differential expression under a bud set experiment in which plants were transferred from constant light to short day conditions (Källman 2009). Of these loci, *PaCCA1*, *PaCDF1*, and *PaAP2L3* also show similarity to flowering time genes. The length of the genes ranges from 1.5 kb to 7.2 kb, with a mean of 3.7 kb.

**Figure 1:**
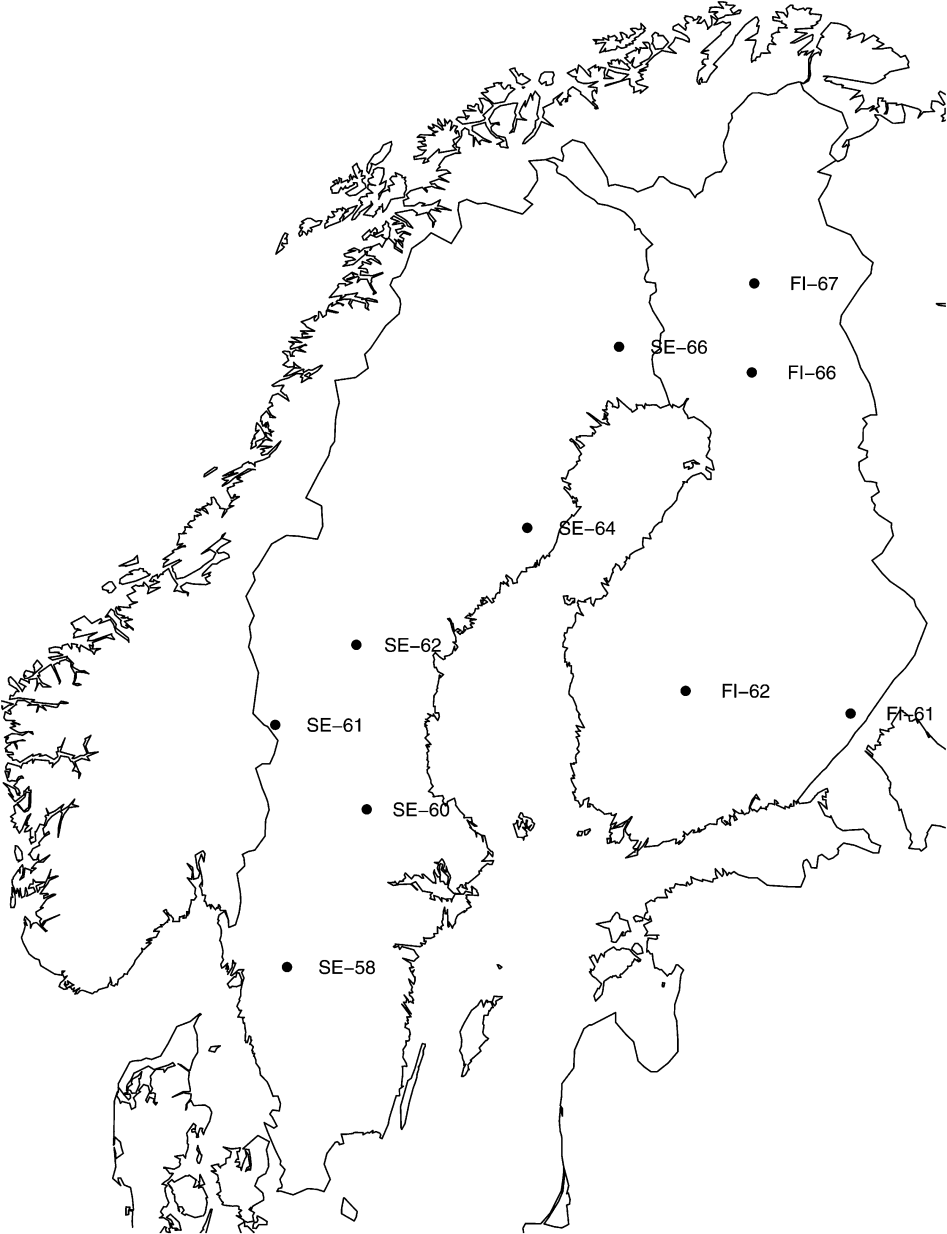
Map of Scandinavia with the locations of sampled populations.

Ten natural populations from the Scandinavian distribution of *P. abies* were chosen along a latitudinal gradient on both sides of the Baltic Sea, ranging from 58°N to 67°N (Figure 1). Seeds from eight trees were sampled from each population except for two Swedish populations, Fulufjället (SE-61) and Höglunda (SE-64), and the most northern Finnish population of Sodankylä (FI-67), where 24 trees were sampled (Table 1). Total genomic DNA was extracted from the haploid megagametophyte tissue of one seed per individual using DNeasy plant mini kit (QIAGEN). Because the aim was to obtain long-range estimates of LD, we chose to amplify genes of considerable length in full, or as overlapping fragments when total length exceeded successful amplification, and to sequence only the ends of amplified fragments to reduce cost (Voight *et al.* 2005; Berlin *et al.* 2011). A graphical representation of the genes with exon and intron structure was produced using the GSDS server (Guo *et al*. 2007). Amplifications were performed with primer sequences and conditions as described in Supporting Information, Table S1. This paired-end sequencing approach was performed using an ABI3730XL sequencer (Macrogen Inc., Seoul, Korea). Sequences were base-called, assembled, and visualized for manual inspection and editing using the PHRED/PHRAP/CONSED program suite (Ewing *et al.* 1998; Ewing and Green 1998; Gordon *et al.* 1998). Bases with a Phred score >20 were retained for analyses. Sequenced fragments were aligned to existing full-length sequences of all genes to determine the length between sequenced ends. An alignment file containing sequence data for all loci and individuals is available as File S1.

**Table 1:** Location of the populations used in this study and sample size

Population	Name	Latitude	Longitude	No. Sampled Individuals
Saleby	SE-58	58° 36′N	13° 12’E	8
SörAmsberg	SE-60	60° 45′N	15° 42’E	8
Fulufjället	SE-61	61° 57′N	12° 78’E	24
Strängsund	SE-62	62° 63′N	15° 12’E	8
Höglunda	SE-64	64° 08′N	18° 74’E	24
Jock/Erkinvinsa	SE-66	66° 58′N	22° 70’E	8
Punkaharju	FI-61	61° 72′N	29° 39’E	8
Vilpuula	FI-62	62° 02′N	24° 63’E	8
St2	FI-66	66° 24′N	26° 53’E	8
Sodankylä	FI-67	67° 41′N	26° 62’E	24

### Population structure

For each locus, Arlequin v 3.5 (Excoffier and Lischer 2010) and R (R Development Core Team 2012) were used to identify linked SNPs with a significant *r^2^* of > 0.2 after Bonferroni correction. These SNPs were subsequently removed from a multilocus data set of all haplotypes and the population genetic structure was investigated using STRUCTURE v 2.3.2 (Pritchard *et al.* 2000). All 11 genes and all individuals (in total 255 SNPs) were used in one analysis whereas a more stringent approach, excluding 15 individuals for which only a few genes were successfully sequenced and filtering loci with more than 15% missing data, left loci from seven genes represented by 112 SNPs to be analyzed. For both data sets, we used the LOCPRIOR model with correlated allele frequencies among populations to perform the analysis for a number of clusters ranging from K = 2 to K = 10 with 10 independent runs for each K. A burn-in period of 100,000 iterations and a run length of 1,000,000 iterations were used. We merged data from the 10 runs using the software CLUMP (Jakobsson and Rosenberg 2007). Global F_ST_ was calculated between the 10 populations for each locus in DnaSP v 5.10 (Librado and Rozas 2009).

### Nucleotide diversity and sampling effect on Tajima’s D

Standard population genetics parameters were calculated using DnaSP v 5.10 (Librado and Rozas 2009) for the combined data set as well as for each of the three populations with larger sample size. Indels were excluded from all analyses. Tajima’s D was computed for all 10 populations to evaluate deviations from the standard neutral model. To examine the effect of sample size on estimates of neutrality in *P. abies*, eight individuals were resampled randomly from each of the three populations with larger sample size. The resampling was repeated 100 times in each population and COMPUTE (Thornton 2003) was used to calculate Tajima’s D for each of the resampled data sets, excluding missing data to get comparable results to those obtained with DnaSP v 5.10 (Librado and Rozas 2009).

### Levels of LD

The squared correlation of allele frequencies (*r^2^*; Hill and Robertson 1968) and D′ (Lewontin 1964) were used to estimate the degree of LD per locus and calculated based on pairwise comparisons between informative sites only in DnaSP v 5.10 (Librado and Rozas 2009). The two measures of LD provide different information about the association between alleles: *D′* reflects recombination between the two loci whereas *r*^2^ is more informative on the power to predict allele identities at one locus given allelic states at another locus. Fisher’s exact test was used to determine the statistical significance of each pairwise test at a level of *P* < 0.05 after Bonferroni’s correction. The decay of LD over distance was investigated by plotting *r^2^* values between all informative sites against the distance between sites and fitting the expectation of *r^2^* to the observed data, applying the formula from Hill and Weir (1988) in R (R core team 2012). The same was done with D′. In this case the decay of D′ was fitted to the function D′(t) = (1-r)^t^ where r is the recombination fraction between pairs of SNP and t, the parameter to be estimated, is the number of generations since D′ = 1. In order to compare patterns over populations we assumed 1 cM = 1 Mb. However, because there are no data available on the relationship between genetic distance (centiMorgan) and physical distance (base pairs) for spruce species we also performed the analysis assuming 1 cM = 10 Mb, 1 cM = 20 Mb and 1 cM = 0.5 Mb, but because the comparisons among populations were not altered we only report results for 1 cM = 1 Mb. R code written by F. Marroni and available at http://fabiomarroni.wordpress.com/ was used to estimate t. The results were computed for each gene separately and for the complete data set. Finally, all genes were used to investigate the decay of LD within each of the populations with a larger sample size.

### Recombination

The composite likelihood method implemented in LDhat (McVean *et al.* 2002) was used to estimate the population recombination parameter ρ = 4N_e_r for each locus, where N_e_ is the effective population size and *r* is the per locus recombination rate per generation. Because our data set contained missing data, we took advantage of the precalculated likelihood files that are included with the software and assumed a fixed θ = 4N_e_μ οf 0.001 for all loci. The per-locus estimate was calculated using the data set including all population, and using the three populations of larger sample size separately.

## Results

**Figure 2:**
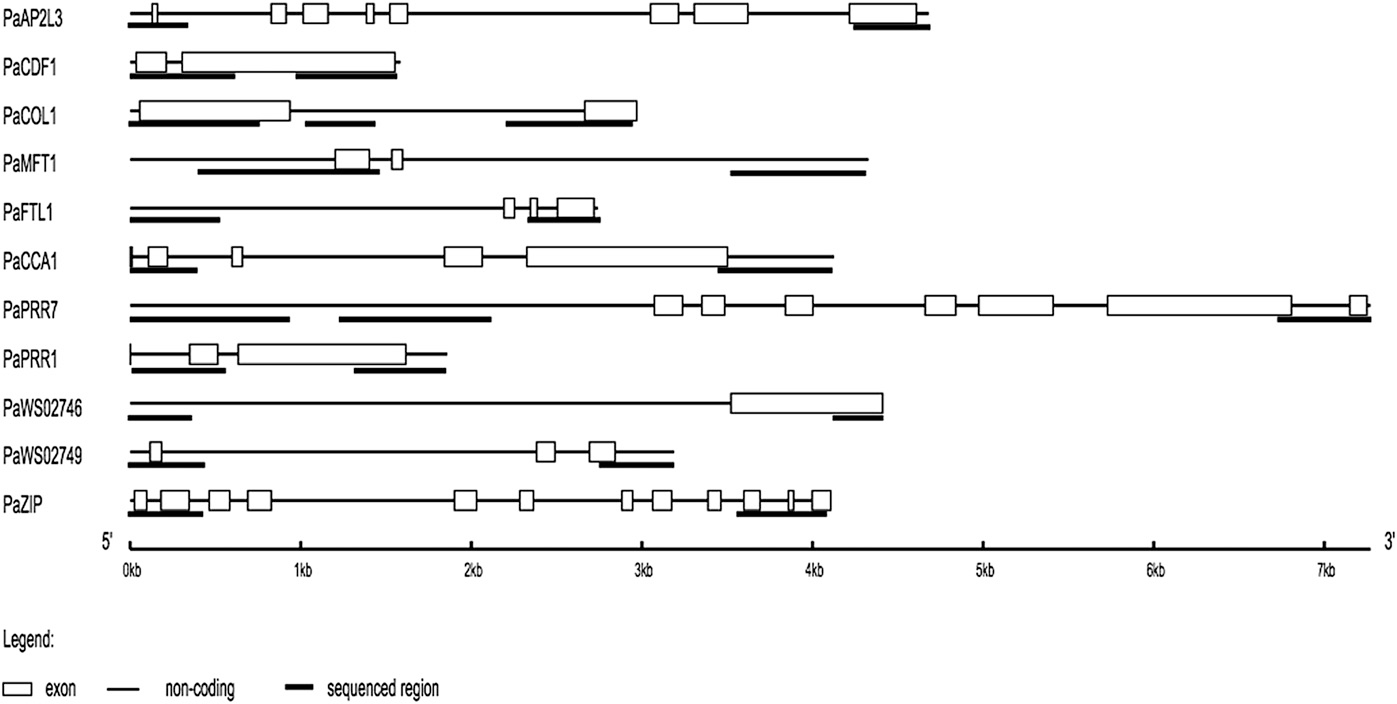
A schematic representation of the eleven genes amplified in this study. The regions sequenced and analyzed are indicated underneath each gene (see Legend).

Based on the individuals with shortest read length in every locus, a total of ~1.11 Mbp (1,118,584 bp) of sequence was used for the estimation of population genetics parameters (Table 2). A schematic representation of the genes and the regions screened for variation can be found in Figure 2. On average, 97 individuals per gene were successfully sequenced (ranging from 74 to 114 individuals) and 1062 bp were sequenced per gene (ranging from 457 to 2449 nucleotides), yielding ~103,400 bp of sequence data per gene. For analyses allowing for missing data the full range of read lengths among individuals was used, increasing the amount of sequence data analyzed. In the restricted dataset, we identified a total of 356 segregating sites, of which 142 were singletons and 209 were parsimony informative sites. Five sites were found to be multi allelic and were not included in the analyses.

**Table 2:** Nucleotide diversity and summary statistics for the 11 loci used to estimate long-range LD and structure in populations of *P. abies*

Gene	N	Length of Amplicon, bp	Bp Sequenced	S (Singletons)	H	Hd	θw	π	Tajima’s D
PaAP2L3	74	4681	457	5 (1)	7	0.58	2.2	1.8	−0.42
PaCDF1	107	1585	1028	23 (4)	22	0.92	4.3	2.9	−0.94
PaCOL1	81	2970	2449	64 (26)	39	0.97	5.3	3.3	−1.22
PaMFT1	96	4328	1597	62 (34)	54	0.95	7.6	3.3	−1.81*^a^*
PaFTL1	109	2742	748	14 (4)	14	0.82	3.6	3.3	−0.18
PaCCA1	88	4126	742	24 (5)	21	0.90	6.4	4.1	−1.1
PaPRR7	93	7271	1796	31 (21)	23	0.88	3.4	1.6	−1.65
PaPRR1	114	1859	986	25 (8)	20	0.89	4.8	4.8	0.02
PaWS02746	97	4411	470	34 (13)	40	0.96	14.1	12	−0.43
PaWS02749	100	3189	605	53 (20)	23	0.82	16.9	10.5	−1.21
PaZIP	113	4107	803	21 (6)	15	0.74	4.9	3.6	−0.78

LD, linkage disequilibrium; N, Sample size; S, Number of segregating sites; H, Number of observed haplotypes; Hd, Observed haplotype diversity; θw, Watterson’s estimate of θ (×10^-03^); π, Average nucleotide diversity (×10^-03^).

a Significant deviation from the standard neutral model.

In the three populations of larger sample size, the average number of sequenced individuals was 20 in SE-61 and 16 in both SE-64 and FI-67. The average length of sequence analyzed per gene was 1224 bp in SE-61, 1251 bp in SE-64, and 1393 bp in SE-67, with a total of 218, 187 and 208 segregating sites identified respectively, for details see Table 3.

**Table 3:** Summary sequence statistics for the 11 loci within the populations SE-61, SE-64, and FI-67

									Resampling	
Gene	Pop	N	Bp	S	Hd	θw	π	Tajd	Mean π (min, max)	Mean TajD (min, max)
PaAP2L3	SE-61	16	519	5 (2)	0.69	2.9	2.4	−0.53	1.2 (0.5, 1.6)	−0.28 (−1.0, 0.3)
	SE-64	7	544	4 (0)	0.67	3	3.9	−1.35	n.a.	n.a.
	FI-67	16	811	7 (0)	0.77	2.6	3.3	0.99	2.5 (1.4, 3.1)	0.57 (−0.7, 1.4)
PaCDF1	SE-61	20	1179	15 (7)	0.94	3.6	2.6	−1.03	2.0 (1.3, 2.8)	−0.51 (−1.3, 0.5)
	SE-64	18	1506	11 (2)	0.84	2.1	2.4	0.47	2.2 (1.4, 2.7)	0.13 (−0.5, 1.2)
	FI-67	16	1330	14 (6)	0.94	3.2	2.6	−0.76	1.5 (0.8, 2.3)	−0.93 (−1.6, −0.6)
PaCOL1	SE-61	20	2495	40 (15)	0.97	4.5	3.3	−1.11	3.2 (2.9, 3.5)	−0.96 (−1.4, −0.3)
	SE-64	14	2527	30 (15)	0.96	3.7	3.3	−0.54	3.2 (2.5, 4.1)	−0.22 (−0.7, 0.3)
	FI-67	11	2492	23 (4)	0.93	3.2	3.5	0.51	3.5 (3.1, 3.8)	0.42 (−0.3, 1.5)
PaMFT1	SE-61	21	1670	27 (15)	0.96	4.5	3.6	−0.82	3.1 (1.8, 3.9)	−0.18 (−0.8, 0.4)
	SE-64	14	1674	23 (13)	0.99	4.3	3.9	−0.4	3.2 (2.7, 3.6)	0.09 (−0.4, 1.0)
	FI-67	14	1676	15 (7)	0.93	2.8	2.8	−0.1	2.7 (1.9, 6.2)	0.23 (−0.4, 1.5)
PaFTL1	SE-61	20	784	8 (1)	0.77	2.9	2.7	−0.18	1.6 (1.2, 2.0)	−0.2 (−1.0, 0.5)
	SE-64	19	865	11 (4)	0.89	3.6	3	−0.62	1.7 (1.0, 2.6)	−0.49 (−1.3, 0.7)
	FI-67	17	1206	14 (6)	0.82	3.4	3	−0.52	2.3 (1.1, 3.8)	−0.38 (−0.9, 0.5)
PaCCA1	SE-61	20	992	16 (6)	0.89	4.6	3	−1.23	2.1 (1.3, 2.9)	−0.85 (−1.3, 0.2)
	SE-64	15	900	14 (5)	0.93	4.8	3.7	−0.94	2.1 1.3, 2.7)	−1.01 (−1.5, 0.2)
	FI-67	15	896	17 (11)	0.93	5.8	3.4	−1.69	1.8 (1.1, 2.8)	−1.29 (−1.8, −0.4)
PaPRR7	SE-61	18	2477	18 (9)	0.95	2.1	1.6	−0.95	1.2 (0.9, 1.7)	−0.45 (−1.4, 0.2)
	SE-64	19	2513	15 (8)	0.9	1.7	1.2	−1.06	1.5 (0.6, 1.8)	0.00 (−0.8, 0.4)
	FI-67	18	3178	24 (13)	0.94	2.2	1.6	−1.07	1.2 (1.0, 1.4)	−0.63 (−1.0, −0.2)
PaPRR1	SE-61	22	1068	21 (10)	0.94	5.4	4.6	−0.53	4.1 (2.4, 5.4)	0.22 (−0.3, 1.1)
	SE-64	18	1054	18 (7)	0.86	5	5.6	0.49	4.0 (2.1, 5.8)	−0.01 (−1.1, 0.9)
	FI-67	21	1035	16 (4)	0.89	4.3	5	0.61	3.6 (2.7, 4.8)	0.61 (−0.1, 1.5)
PaWS02746	SE-61	18	515	26 (12)	0.98	15	14	−0.23	3.8 (2.8, 6.5)	−0.33 (−0.8, 0.6)
	SE-64	15	513	19 (7)	0.97	11	12	0.14	4.3 (3.1, 5.9)	0.47 (−1.1, 1.5)
	FI-67	15	959	29 (12)	0.99	9.3	10	0.37	7.4 (6.3, 9.3)	0.37 (−1.0, 1.0)
PaWS02749	SE-61	19	641	28 (14)	0.73	13	8.3	−1.31	4.4 (1.8, 6.4)	−1.04 (−1.8, −0.5)
	SE-64	14	735	28 (13)	0.92	12	9.9	−0.75	5.1 (3.0, 8.6)	−0.58 (−1.5, 0.5)
	FI-67	16	847	28 (17)	0.87	10	8.3	−0.69	5.2 (4.5, 6.3)	−0.30 (−0.8, 0.2)
PaZIP	SE-61	22	1123	14 (3)	0.68	3.4	3.6	0.17	2.6 (1.4, 4.5)	−0.53 (−1.8, 1.2)
	SE-64	22	932	14 (5)	0.8	4.1	3.3	−0.68	2.3 (1.5, 3.2)	−0.57 (−1.7, 1.4)
	FI-67	16	900	21(12)	0.78	4	5.2	1.12	3.1 (2.6, 3.9)	1.09 (−0.3, 2.0)

N, sample size; S, number of segregating sites; Hd, observed haplotype diversity; θw, Wattersons estimate of θ (×10^-03^); π, average pairwise distance (×10^-03^); TajD, Tajima’s D; n.a., not calculated due to low sample size.

### Population structure

There was no clear population structure among the populations sampled in this study. The ΔK method (Evanno *et al.* 2005) suggests that the data set most likely consists of two clusters, whereas the likelihood values suggest that the data set is made up of five, seven, or eight clusters with generally small differences in likelihood between different K values. Plotting individual cluster assignments for both K = 2 and K = 3 (the number of possible clusters suggested by Chen *et al.* 2012) further vindicate the lack of meaningful structure as most individuals look admixed and there is no clear geographic pattern emerging (Figure S1). There was no difference between using all loci (including those with missing data) and the more conservative approach where less missing data were allowed.

In line with these results, observed global F_ST_ values at individual genes between all populations varied between virtually 0 and 0.049. Using only the three populations with larger sample size led to a slight increase in F_ST_ values and for *PaFTL1* an F_ST_ of close to 0.2 was obtained (Table S2).

### Nucleotide diversity and Tajima’s D

**Figure 3:**
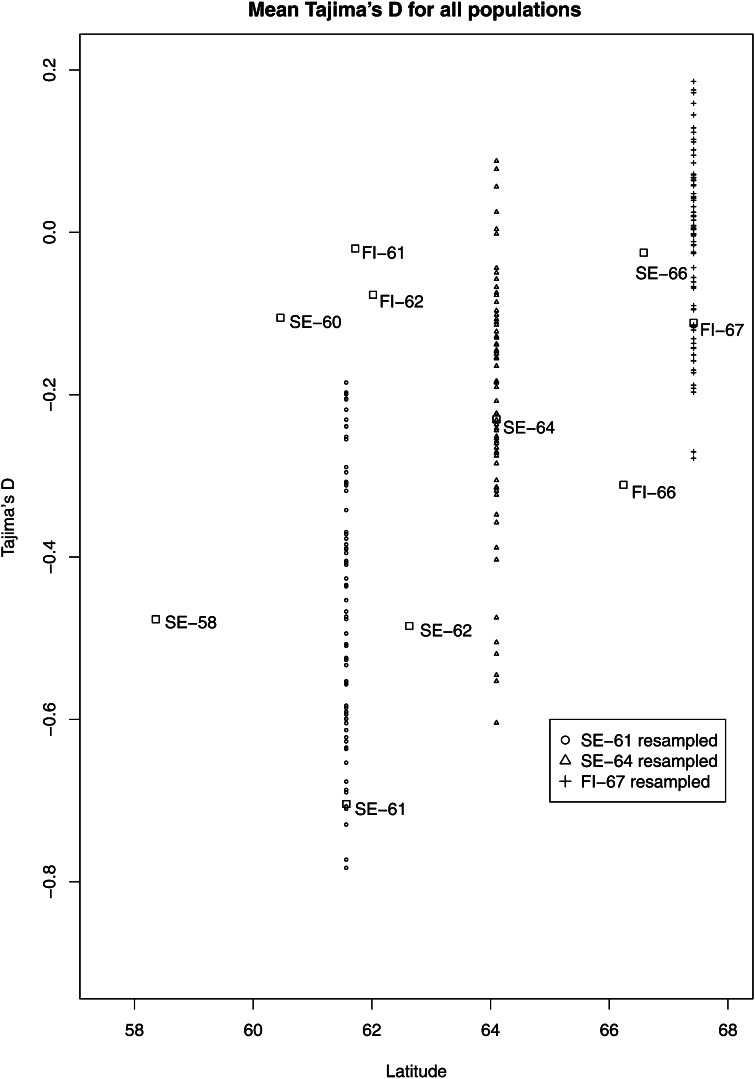
Within population estimates of mean Tajima’s D across eleven loci plotted against latitude of origin. Boxes denote estimates from the number of individuals sampled from the population. The mean across eleven loci within resampled populations SE-61, SE-64 and FI-67 is plotted with circles, triangles and crosses respectively (see legend).

Polymorphisms from both coding and noncoding regions of the genes were used to estimate nucleotide diversity. We chose to analyze them jointly because the amount of coding sequence was limited and treating synonymous and nonsynonymous sites separately would have led to estimates based on only a small number of sites. For the total data set, the mean nucleotide diversity (π) was 0.0047 (SD = 0.0034), the mean value of Watterson’s estimator (θ) was 0.0067 (SD = 0.0046), and the average haplotype diversity was 0.86 (SD = 0.16). Overall, *PaAP2L3* was the least variable gene, with only five polymorphic sites. *PaWS02746* and *PaWS02749* both had greater π and θ values than all other genes but similar values of haplotype diversity, whereas *PaZIP* had lower haplotype diversity with average values of nucleotide diversity. The mean Tajima’s D was −0.88 (SD = 0.59), with *PaPRR1* being the only gene with a positive value (Table 2). The only significant Tajima’s D, −1.81, was found in *PaMFT1*. Mean Tajima’s D over all genes was plotted for each population against latitude of origin, revealing a weak trend toward more positive values as one moves north. The pattern was clear when looking at the three populations with larger sample size, but for the populations with fewer individuals there was no such pattern (Figure 3). Reducing the data set to include only candidate genes for bud set did not alter the outcome (data not shown).

Despite an added amount of sequence data and reduced sample size, the mean π in populations FUL, HOG, and SOD was very similar to the mean obtained over the total data set and single gene estimates varied only slightly between populations (Table 3). In contrast, values of Tajima’s D obtained in the three populations differed from estimates obtained from the total data set and were also quite variable among populations. Several genes had a positive Tajima’s D, with SOD being the population with the most positive values.

### Effect of sample size

As expected, resampling showed that estimates of π were fairly robust to variation in sample size. In contrast, Tajima’s D showed large fluctuations across the resampled data sets. In some instances, the mean of the 100 resampled estimates was close to the estimate based on all individuals in the same population, but in many instances the estimated Tajima’s D value was even reversed, shifting from negative to positive or vice versa. The largest difference in values of Tajima’s D was for *PaZIP* in HOG, where resampled population values ranged from −1.74 to 1.43 (Table 3).

### Levels of LD and recombination

**Figure 4:**
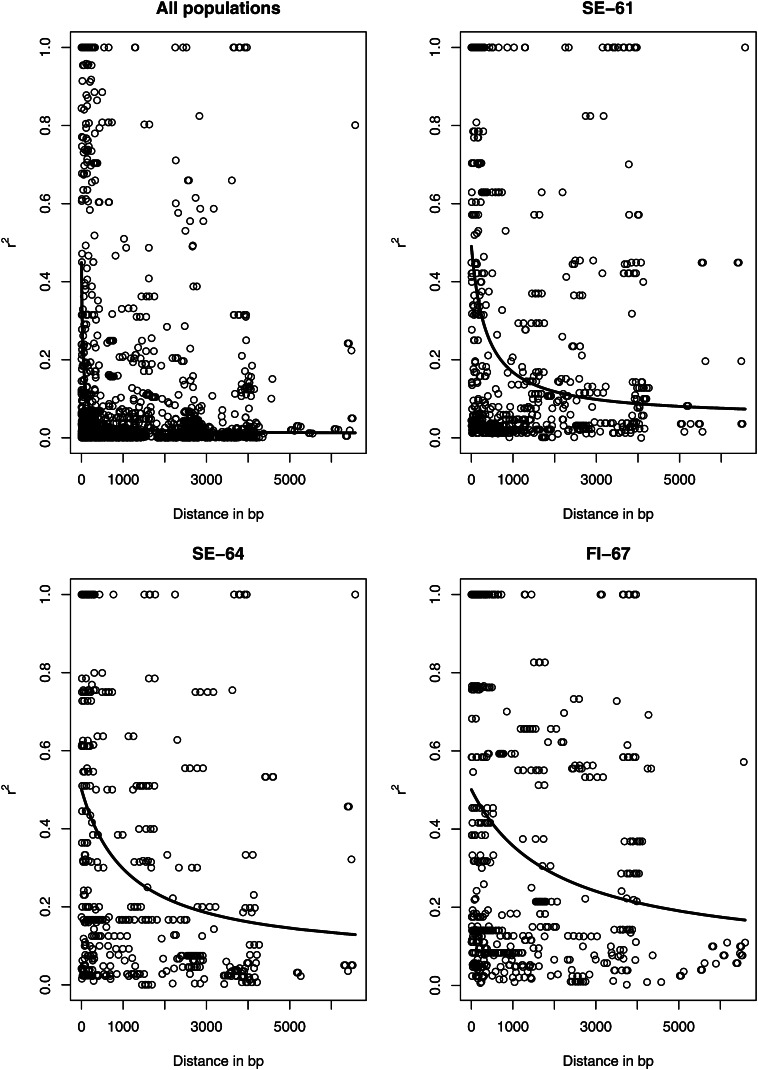
Plot of the squared correlation of allele frequencies (r^2^) *vs.* distance in base pairs across 11 loci for different subsets of populations. (Top left) all ten populations n=97, (top right) SE-61 n=20, (bottom left) SE-64 n=16, and (bottom right) FI-67 n=16.

The informative sites of all 11 genes generated 2378 pairwise comparisons, 272 (11.4%) of which remained significant after Fisher’s exact test with Bonferroni’s correction. The average *r^2^* over all pairwise comparisons was 0.11 (SD = 0.05) and LD decreased rapidly with distance between pairs of SNPs, reaching an *r^2^* value of less than 0.2 within 80 bp (Figure 4A). The decrease was slower when D′ was considered (Figure S2).

*PaAP2L3*, with only six pairwise comparisons between informative sites, was excluded from the per gene estimates of *r^2^*. The remaining 10 genes, with 45 pairwise comparisons or more, had mean *r^2^* values from 0.04 in *PaCDF1* to 0.2 in *PaZIP*, with five genes having a mean *r^2^* > 0.11 (Table 4). The mean *r^2^* value was 0.15 for the above-average group of genes and 0.08 for the below-average group, with *r^2^* values being differently distributed in the two groups (Wilcoxon’s rank sum test: *P* < 0.01). There was no significant correlation between π and mean *r^2^* (Spearman’s rank correlation: r = 0.56, *P* = 0.07). The decay of LD with distance varied between genes, again with *PaCDF1* in the low extreme, where the fitted curve never reached an *r^2^* value of 0.2, and *PaZIP* standing out as LD extended for 353 bp before declining to values less than 0.20. Roughly, the genes can also be divided into two groups based on their pattern of LD. The first consists of *PaCCA1*, *PaCDF1*, *PaCOL1* and *PaWS02746*, where most SNPs are in weak linkage and few SNPs in high linkage appear at distances greater than 1kb (Figure S3). The other group, including the rest of the genes, also displays a great number of SNPs in weak LD but, in addition, displays SNPs in complete LD at distances of several thousand base pairs and SNPs in high LD throughout the length of the genes (Figure S3).

**Table 4:** Mean linkage disequilibrium and recombination rate parameters estimated per locus for the merged data set of 10 populations with a mean of 97 individuals

	Number of Sites			r2				
Gene	Informative	Pairwise*^a^*	Sign. Pairwise, %*^a^*	Mean	<0.2*^b^*	ρ	ρ/site	ρ/θ
PaAP2L3	4	6	16.7	n.a.	n.a.	2.04	0.0004	1.98
PaCDF1	19	171	6.4	0.041	0	5.10	0.003	1.16
PaCOL1	38	703	6.3	0.079	74	18.4	0.006	1.42
PaMFT1	28	378	15	0.056	12	25.5	0.006	2.11
PaFTL1	10	45	28.9	0.13	5	19.4	0.007	7.29
PaCCA-1	18	153	8.5	0.069	7	11.2	0.003	2.36
PaPRR7	10	45	20	0.097	88	8.16	0.001	1.34
PaPRR1	17	136	30.9	0.17	96	3.06	0.002	0.65
PaWS02746	19	171	21.6	0.14	118	21.4	0.005	3.24
PaWS02749	31	465	11.8	0.12	59	11.2	0.004	1.10
PaZIP	15	105	26.7	0.2	353	0	0	0
All Loci	209	2378	11.4	0.1	46	11.4	0.003	1.81

n.a., not applicable.

a Number of pairwise comparisons and the fraction of these that are significant.

b Number of base pairs where estimated r2 falls below 0.2.

The recombination estimate ρ per site ranged from 0 in *PaZIP* to 0.0071 in *PaFTL1*, with a mean value for all loci of 0.0033 (Table 4). The likelihood curves indicate good estimates of ρ in all genes except *PaCOL1*, *PaFTL1*, *PaMFT1*, and *PaWS02746* (Figure S4). There was no significant correlation between ρ per site and mean *r^2^* (Spearman’s rank correlation: r = −0.3, *P* = 0.34). The estimate of ρ over θ was >1 for all loci except *PaPRR1* and *PaZIP* (Table 4).

Mean *r^2^* was elevated for all genes in all within population estimates compared to the merged data set (Table 5). A multilocus estimate of the decay of LD within populations was strikingly different from the merged data set, with LD extending from 705 bp in SE-61 to 4580 bp in FI-67 before falling under *r^2^* values of 0.2 (Figure 4, B−D). The difference was not as striking when the decay of D’ was considered (Figure S2). The population recombination rates varied greatly, but likelihood curves for the smaller sample sizes were not showing distinct peaks at estimated values (Figure S4).

**Table 5:** Mean linkage disequilibrium and recombination rate parameters estimated per locus for each of the three populations SE-61, SE-64, and FI-67

		Number of Sites			r2				
Gene	Pop		Informative	Pairwise*^a^*	Sign. Pairwise, %*^a^*	Mean	<0.2*^b^*	ρ	ρ/site	ρ/θ
PaAP2L3	SE-61		3	3	0	−0.16	n.a.	2.04	0.4	1.35
	SE-64		4	6	0	−0.69	n.a.	10.2	2.2	6.25
	FI-67		7	21	9.5	0.32	n.a.	2.04	0.4	0.97
PaCDF1	SE-61		8	28	3.6	0.14	n.a.	6.12	3.9	1.45
	SE-64		9	36	8.3	0.25	n.a.	0	0	0
	FI-67		8	28	0	0.15	n.a.	13.3	8.4	3.14
PaCOL1	SE-61		25	300	0	0.16	n.a.	23.5	7.9	2.08
	SE-64		15	105	0	0.29	n.a.	12.2	4.1	1.3
	FI-67		19	171	0	0.37	n.a.	4.08	1.4	0.52
PaMFT1	SE-61		11	55	10.9	0.25	n.a.	20.4	4.7	2.72
	SE-64		10	45	13.3	0.37	n.a.	2.04	0.5	0.28
	FI-67		8	28	21.4	0.44	n.a.	3.06	0.7	0.65
PaFTL1	SE-61		7	21	9.5	0.29	n.a.	7.14	2.6	3.17
	SE-64		7	21	4.8	0.2	n.a.	2.04	0.7	0.65
	FI-67		8	28	14.3	0.34	n.a.	8.16	3	1.97
PaCCA1-l	SE-61		10	45	2.2	0.19	n.a.	7.14	1.7	1.58
	SE-64		8	28	0	0.2	n.a.	0	0	0
	FI-67		6	15	0	0.29	n.a.	0	0	0
PaPRR7	SE-61		9	36	5.6	0.22	n.a.	3.06	0.4	0.58
	SE-64		7	21	9.5	0.2	n.a.	3.06	0.4	0.71
	FI-67		11	55	1.8	0.16	n.a.	5.1	0.7	0.73
PaPRR1	SE-61		11	55	12.7	0.29	n.a.	2.04	1.1	0.35
	SE-64		11	55	23.6	0.52	n.a.	0	0	0
	FI-67		12	66	16.7	0.37	n.a.	2.04	1.1	0.46
PaWS02746	SE-61		14	91	17.6	0.38	n.a.	8.16	1.9	1.08
	SE-64		12	66	6.1	0.32	n.a.	18.4	4.2	3.14
	FI-67		16	120	23.3	0.5	n.a.	12.2	2.8	1.37
PaWS02749	SE-61		14	91	11	0.39	n.a.	3.06	1	0.38
	SE-64		15	105	0	0.37	n.a.	2.04	0.6	0.23
	FI-67		11	55	29.1	0.55	n.a.	7.14	2.2	0.85
PaZIP	SE-61		11	55	40	0.54	n.a.	0	0	0
	SE-64		9	36	58.3	0.55	n.a.	0	0	0
	FI-67		9	36	44.4	0.66	n.a.	0	0	0
Mean	SE-61		123	780	8.6	0.261	705	7.5	2.3	1.34
	SE-64		107	524	9.5	0.348	2549	4.5	1.2	0.88
	FI-67		115	623	13.5	0.398	4580	5.2	1.9	0.94

n.a., not applicable.

a Number of pairwise comparisons and the fraction of these that are significant.

b Number of base pairs where estimated r2 falls below 0.2.

## Discussion

### Population structure

Despite of the fact that the populations covered up to 9º of latitude (from 58° 36′N to 67° 41′N) we were unable to detect a clear genetic structure among the populations sampled within the Scandinavian distribution of *P. abies*. This confirms the lack of structure generally observed in central Scandinavia in Norway spruce, but is slightly different from the results of Chen *et al.* (2012) where populations above 67°N tended to form a separate cluster. The lack of population structure here could simply reflect a lower power in the present study than in Chen *et al.* (2012) and the presence of a smaller number of populations at high latitudes in this study.

### Nucleotide diversity and Tajima’s D

The overall level of nucleotide diversity was for the most part comparable with previous results in *P. abies* (Heuertz *et al.* 2006; Chen *et al.* 2010), with greater values for *PaWS02746* and *PaWS02749* and its estimate was not sensitive to sampling. The mean Tajima’s D was negative, again consistent with previously reported results and an expanding population size (Heuertz *et al.* 2006; Chen *et al.* 2010). Because we did not detect any meaningful population structure, the overall estimate of Tajima’s D is unlikely to have been strongly affected by pooling of samples from different populations (Städler *et al.* 2009) and given the size of the total sample the estimate of Tajima’s D should be close to its true value (Marroni *et al.* 2011). In line with the results of Marroni *et al.* (2011) and in contrast to estimates of π, estimates of Tajima’s D value and its significance were quite sensitive to the reduction in sample size and, apart from the general trend of an increase in positive values associated with smaller sample sizes, resampling displayed a wide range of values depending on the randomly sampled representatives of the population. The trend of more negative values of Tajima’s D with increasing number of individuals is contrary to neutral expectations under a fixed number of segregating sites, where adding individuals should cause Tajima’s D to become more positive. However, as Norway spruce reveals signs of population expansion throughout the distribution range (Heuertz *et al.* 2006; Chen *et al.* 2010), increasing the sample size in this species, in effect, means introducing new mutations occurring in low frequencies or even as singletons, leading to more negative values of Tajima’s D and a more accurate reflection of the demographic history of Norway spruce. It seems, therefore, that estimates of Tajima’s D based on small samples (<50 according to Table 5 in Marroni *et al.* 2011) should be considered with caution, especially in populations that putatively depart from the standard neutral model and harbor a large number of rare variants. A striking example of the increase of the number of segregating sites with increased sample size is provided by the recent study of Nelson *et al.* (2012) in humans, where θ_W_, which is based on S, increased fivefold when sample size went from 500 to 10,000 whereas θ_π_ remained stable. Finally, as we would expect sampling to affect Tajima’s D more strongly when populations are structured and depart from the standard neutral model, resampling can in itself be a new source of information on past demographics that warrants further investigation (Cutter *et al.* 2012; St. Onge *et al.* 2012).

We were unable to detect a clear latitudinal gradient of Tajima’s D values among the 10 populations, but as resampling clearly showed, a sample size of eight individuals was not sufficient for this data set to yield robust estimates of Tajima’s D within populations. The three populations with a larger sample size did reflect a pattern of more positive values of Tajima’s D with increased latitude similar to the pattern observed in *P. sitchensis* (Holliday *et al.* 2010), but with only three populations of sufficient sample size it is difficult to accurately determine if the pattern is true. Obviously, to assess reliably the presence of a latitudinal cline in Tajima’s D, a larger number of individuals per population as well as a larger number of loci would be required.

### Level of LD and recombination

**Figure 5:**
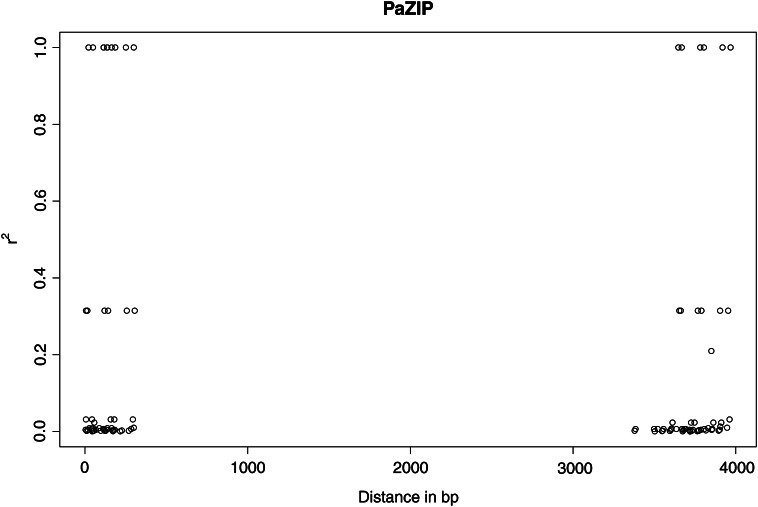
Plot of the squared correlation of allele frequencies (r^2^) *vs.* distance in base pairs in the gene *PaZIP* using all populations.

The within gene level of LD in this study was generally low. However, *P. abies* does not seem to be an exception to the pattern of LD varying between genes shown in other conifers. Estimating the decay of LD with distance using the pooled *r^2^* estimates for all genes failed to reflect the heterogeneous levels of LD among the genes, evident in the variable estimates of linkage and recombination across genes. *PaZIP* and *PaPRR1* in particular displayed high mean *r^2^* values. Plotting the squared correlation of allele frequencies against distance in *PaZIP* reveals that SNPs in complete LD extend throughout the length of the gene, a total of 4 kb (Figure 5), resembling the pattern found for allozyme coding loci in pine (Pyhäjärvi *et al.* 2011). More sequence data may reveal a consistent pattern of LD along the whole length of *PaZIP*. In *Pinus pinaster*, a gene coding for transcription factor from the HD-ZIP family, *HD-ZIPIII*, showed high levels of LD across more than 3.2 kb (Lepoittevin *et al.* 2012).

For *PaZIP*, the high mean *r^2^* value of 0.2 was matched by a population recombination rate of 0, but contrary to expectations this was not a general occurrence because we could not find a significant correlation between mean *r^2^* and ρ per site across genes. The flat likelihood curves for four of the genes, indicating some uncertainty in the estimate of ρ, may explain the lack of correlation. Less surprising was the lack of correlation between mean *r^2^* and π, as previous studies have obtained the same result in both *P. glauca* and *P. abies* (Pavy *et al.* 2012). The ratio of ρ to θ revealed that recombination was the major force behind the variation in all loci except *PaPRR1* and *PaZIP*, which is consistent with their high mean *r^2^* values and relatively slow decay of LD, maintaining mean *r^2^* values > 0.2 over more than 100 bp.

Despite the lack of population structure between the Scandinavian populations, LD and recombination were quite variable between the three populations more intensely sampled. In part, the inflated values of LD can be explained by the reduced sample size in within population estimates because *r^2^* depends on allele frequencies and an absence of rare alleles will tend to inflate LD estimates slightly (Hedrick and Kumar 2001). However, it seems unlikely that the increased distances of LD decay evident in individual populations should only be a result of a reduction in sample size. In a study on the effect of minor allele frequency thresholds on estimates of LD in *Populus nigra* the decay of LD estimated with *r^2^* increased by a distance of ~400 bp when increasing the threshold from 0.05 to 0.1 (Marroni *et al.* 2011).

Because we only used parsimony informative sites in the calculation of *r^2^*, the minor allele frequency would increase from 0.02 in the merged data set with a mean sample size of 97 individuals to 0.125 in the mean sample size of 16 individuals in FI-67. This does not seem sufficient to explain the increase in distance of LD decay from 46 bp in the merged data set to 4580 bp in FI-67. Perhaps a more plausible explanation to the difference in the extent of LD between the pooled sample and the individual populations is that, despite the apparent lack of population structure, the three subpopulations have had different histories. Boreal forest tree species, and Norway spruce in particular, have had complex histories and have undergone periodic fragmentation and admixture due to glaciation cycles in some parts of their current range. Simulations have shown that LD created through postglaciation admixture can be elevated and will be maintained in species with long generation times (Tachida 2012) and the difference in LD levels between populations may be the result of subtle differences in past fragmentation-admixture events. Partial admixture, or population structure, could also be an explanation as Tachida (1994) in a finite island model showed that the initial rate of decay of LD is increased by finiteness of the population but that the ultimate rate of decay is decreased. Hence, LD created in the past may persist longer in smaller subdivided populations (Tachida 1994). If the populations have a different past then we would expect different levels of LD. We note that there is a cline in LD with the lowest value found in SE-61 and the highest in FI-67 (Figure 4, B−D and Table 5). Although we did not detect a clear differentiation between FI-67 and the other populations in the present study, other studies (X.-R. Wang, personal communication) (Chen *et al.* 2012) have shown that populations above 65°N are genetically different from more southerly Scandinavian populations. The exact cause of this genetic difference is not yet well understood. It could reflect differences in origins and/or differences in selection pressure and reproduction. In any case the extensive LD observed in FI-67 could reflect this difference. A demographic explanation seems also supported by the fact that differences in the decay of D′ was much less striking than for the decay of *r^2^* with distances. As was noted previously D′ reflects recombination between the two loci, whereas *r*^2^ reflects more the gene genealogies (McVean 2001).

To successfully design an association mapping experiment one needs to have detailed knowledge about many basic population genetic parameters of the species of interest. These parameters are for the majority of species still missing. In Norway spruce, despite the relatively low levels of population structure it is evident that there are differences in both pattern of LD and allele frequencies between populations, calling for caution in estimating parameters on species-wide sampling and highlighting the importance of larger sample size within populations to obtain meaningful results. As the pattern of LD largely determines the power of an association study and the existence of population structure can yield an excess of false positives there are strong incentives to obtain better estimates of these fundamental parameters to be able to optimize the design of association experiments. Together with data from other conifers the data put forward here indicate a more heterogeneous pattern in LD than earlier studies have suggested. Hence the classic view of a lack of LD within genes in conifers needs to be reconsidered. Even though gene space is going to remain specifically interesting for association mapping, especially in species with large genomes, with the arrival of full genome data from a single individual we should now focus our efforts on estimating LD and factors that influence LD at full genome level.

## Supplementary Material

Supporting Information
