# Supplementary material for: Distribution of Long-Range Linkage Disequilibrium and Tajima’s D Values in Scandinavian Populations of Norway Spruce (Picea abies)
Source: G3 (Bethesda). 2013 May 1;3(5):795–806. doi: 10.1534/g3.112.005462 (PMC3656727; doi:10.1534/g3.112.005462)
Supplement: Supporting Information [file supp_3_5_795__index.html]

Distribution of Long-Range Linkage Disequilibrium and Tajima’s D Values in Scandinavian Populations of Norway Spruce (Picea abies) — Supporting Information 

# Distribution of Long-Range Linkage Disequilibrium and Tajima’s D Values in Scandinavian Populations of Norway Spruce (*Picea abies*)

## Supporting Information for Larsson *et al.*, 2013

**Files in this Data Supplement:**

- Supporting Information - Figures S1-S4, File S1, and Tables S1-S2 (PDF, 1 MB)
- Figure S1 - Top figure shows the likelihoods of estimated number of clusters (K) obtained with the program STRUCTURE. (PDF, 87 KB)
- Figure S2 - Plot of D' vs. distance in base pairs across eleven loci for different subsets of populations (PDF, 416 KB)
- Figure S3 - Plot of the squared correlation of allele frequencies (r2) vs. distance in base pairs (PDF, 328 KB)
- Figure S4 - Per locus likelihood curves for the estimate of p using all populations, SE-61, SE-64 and FI-67 respectively (PDF, 194 KB)
- Table S1 - Table of primer sequences and PCR conditions for the genes amplified in this study (PDF, 92 KB)
- Table S2 - Estimates of population differentiation between all populations and between the more densely sampled populations SE-61, SE-64 and FI-67 (PDF, 87 KB)
- File S1 - Supporting Data (.zip, 204 KB)
