# Supplementary material for: Distribution of Long-Range Linkage Disequilibrium and Tajima’s D Values in Scandinavian Populations of Norway Spruce (Picea abies)
Source: G3 (Bethesda). 2013 May 1;3(5):795–806. doi: 10.1534/g3.112.005462 (PMC3656727; doi:10.1534/g3.112.005462)
Supplement: Supporting Information [file supp_g3.112.005462_005462SI.pdf]

G3: Genes | Genomes | Genetics

Distribution of long-range Linkage Disequilibrium and Tajima's D values in Scandinavian populations of Norway spruce (*Picea abies*)

Hanna Larsson[*], Thomas Källman[*], Niclas Gyllenstrand[§], Martin Lascoux[*]

[*]Department of Ecology and Genetics, EBC, Uppsala University, 752 36 Uppsala, Sweden

[§]Department of Plant Biology and Forest Genetics, Swedish University of Agricultural Sciences, Uppsala, 750 07 Uppsala, Sweden

Author for correspondence:

Hanna Larsson

Hanna.Larsson@ebc.uu.se, larsson.h77@gmail.com

Department of Ecology and Genetics, EBC, Uppsala University

Norbyvägen 18D, 752 36 Uppsala

Tel: +46 18 471 28 16, FAX: +46 18 471 64 25

DOI: 10.1534/g3.112.005462

File S1

Supporting Data

An alignment file containing sequence data for all loci and individuals is available for download at http://www.g3journal.org/lookup/suppl/doi:10.1534/g3.112.005462/-/DC1.

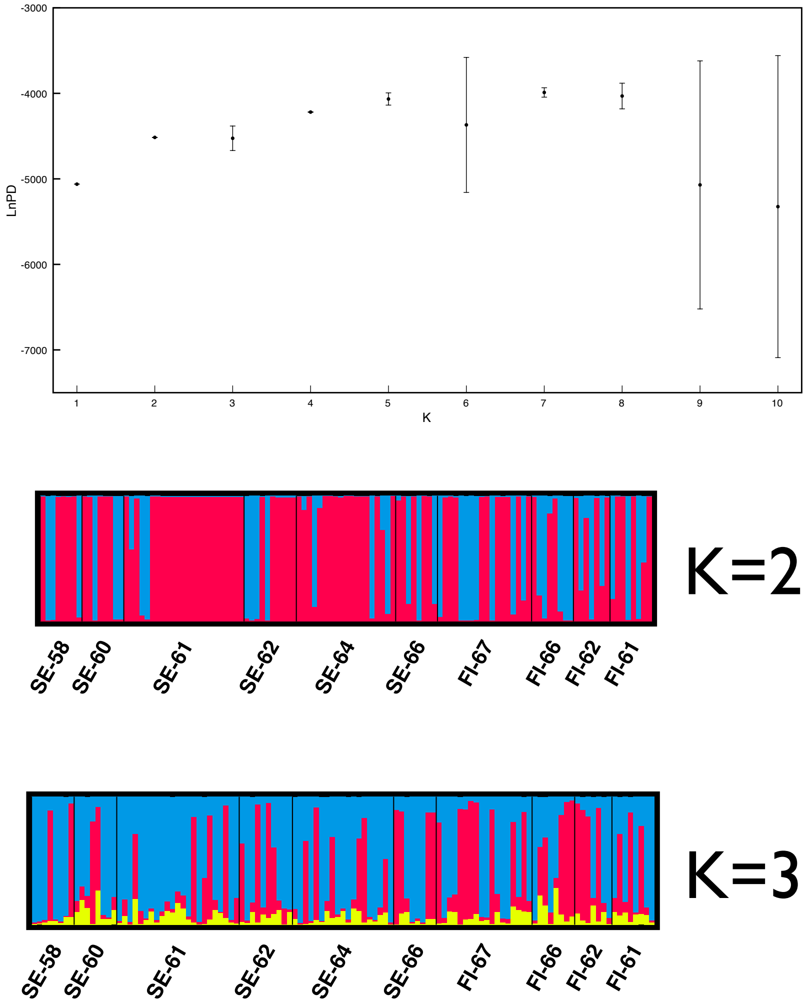

Figure S1 Top figure shows the likelihoods of estimated number of clusters (K) obtained with the program STRUCTURE. The dot represents the mean likelihood over 10 structure runs and the lines represent the standard deviation of the estimate. The bottom two figures show individual assignment results averaged over the ten runs for K=2 and K=3. Note that populations are ordered from south to north in Sweden and then north to south in Finland. The lack of geographic pattern was equally evident for all additional number of clusters tested.

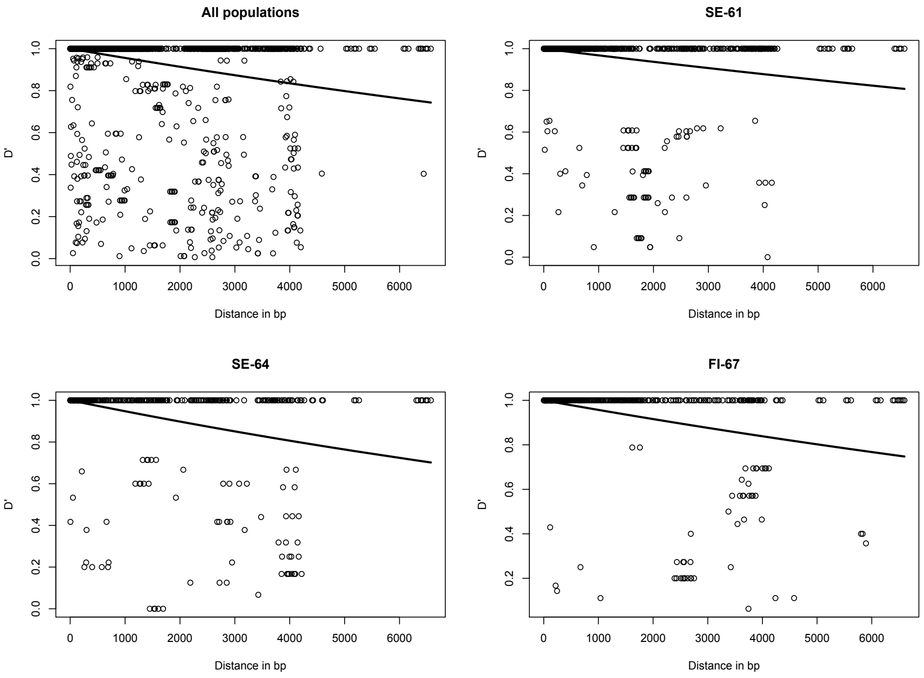

Figure S2 Plot of D´ vs. distance in base pairs across eleven loci for different subsets of populations. From top left to right bottom; a) all ten populations n=97, b) SE-61 n=20, c) SE-64 n=16 and d) FI-67 n=16.

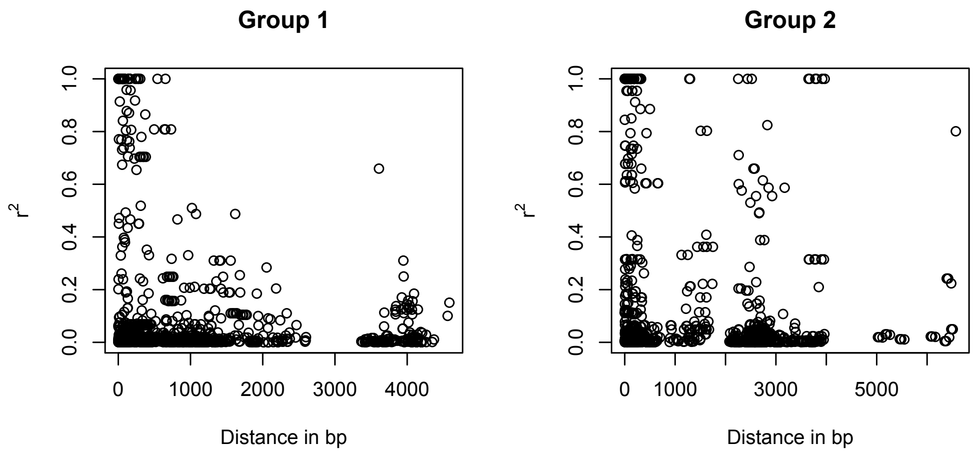

Figure S3 Plot of the squared correlation of allele frequencies (r^2) vs. distance in base pairs. Group 1 consists of the genes *PaCCA1*, *PaCDF1*, *PaCOL1* and *PaWS02746.* Group 2 consists of the genes *PaMFT1, PaFTL1, PaPRR1, PaPRR7, PaWS02749* and *PaZIP*.

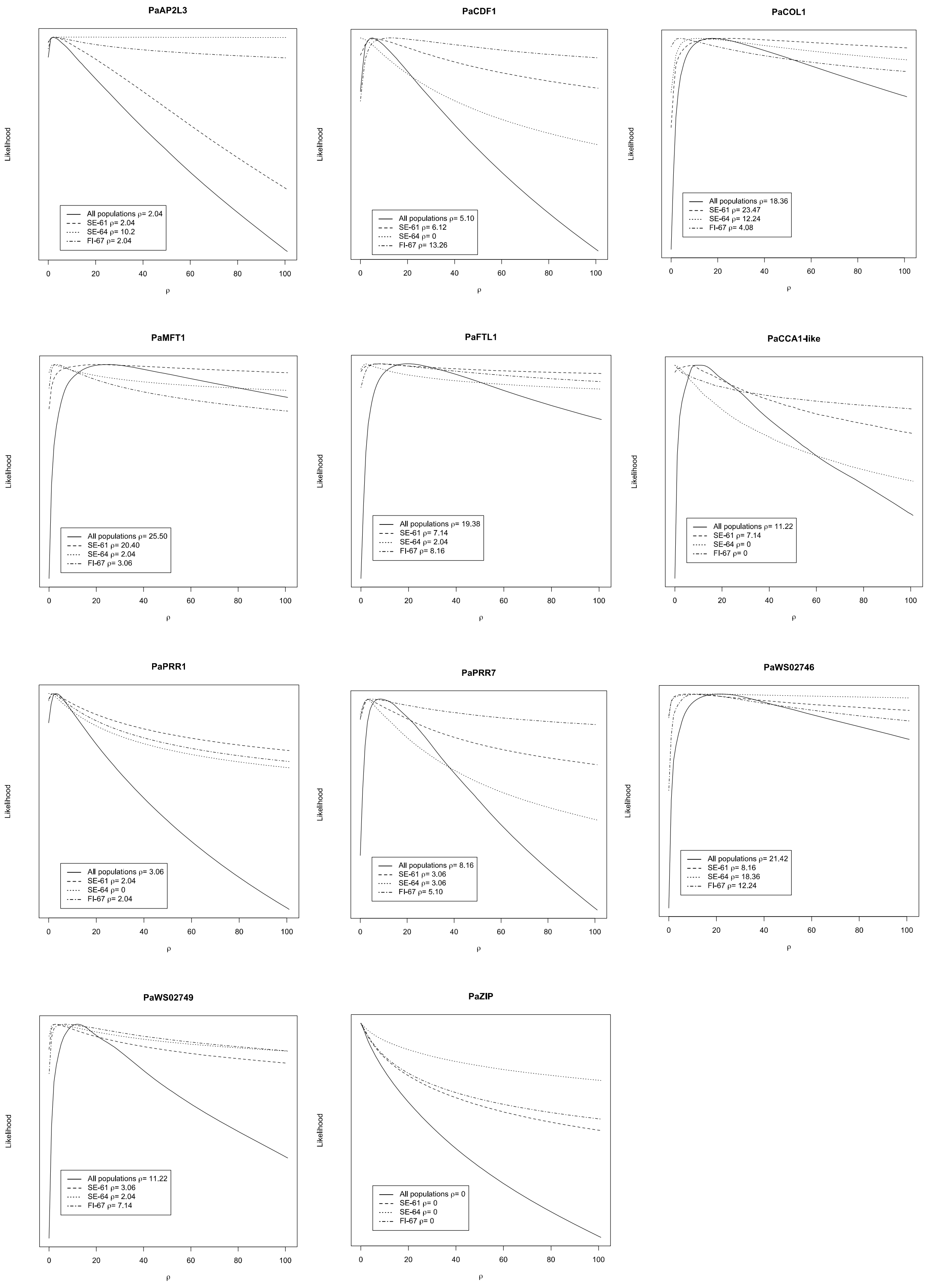

Figure S4 Per locus likelihood curves for the estimate of ρ using all populations, SE-61, SE-64 and FI-67 respectively (see legend). Curves have been rescaled for comparison.

Table S1 Table of primer sequences and PCR conditions for the genes amplified in this study. Multiple primer sequences are given in cases where the gene was amplified in overlapping fragments. All PCRs were run for 35 cycles with an initial heating at 98° for 30 seconds and ending with a 5 minute extension at 72°.

Gene	Forward primer sequence	Reverse primer sequence	Denat.	Ann.	Elong.
PaAP2L3	GGAAACAGGTTTATCTGGGTG	AAGTGACCAAAAGAAAGGCA	98° 10s	60° 20s	72° 3min
PaCDF1	TGTAGAACGGGGTGAGTTG	CTGAACCCTGCTCTTGTAATC	98° 10s	60° 20s	72° 3min
PaCOL1	CAGCAGTGGAGAATGGTGAA	CTGCATCCACATCCAATGAC	98° 10s	60° 30s	72° 30s
	CCACCCTGGTCGACTTTTTA	GGAACAACTCCGTATCCCTGA	98° 10s	60° 30s	72° 3min
PaFT1	CAAGTTCAAATTCAAAGGTAG	GGAGCATCTGGGTCTGTCAT	98° 10s	61° 30s	72° 40s
	AATGTTCGCACCTGGTTTTT	GAAGTGCTCCACAACCAACC	98° 10s	60° 30s	72° 30s
PaFT2	TGAGGACCTTCGCAACTTTT	TGTCTGATTCATTCATGGCTTC	98° 10s	63° 15s	72° 3min
PaCCA1	TATTCTCACTCTCAGCGGGG	GGACAAAACCCACTCCAGACT	98° 10s	63° 15s	72° 3min
PaPRR7	TATAAGGTTAATGAAGGGCTAG	ATAAGATGTGAATGAGAATGAA	98° 10s	59° 30s	72° 1min
	TCTTTTGGGGTAAACAACTCCT	AGAGATACACTGATAGCCTTAC	98° 10s	60° 20s	72° 3min
	AAACCTGTTAGAAAGAACGAGC	AGAGGCAAATTGTAAATATCCC	98° 10s	60° 20s	72° 3min
PaPRR1	GGCCAGTCATCCTGAGTGCGAGTCAC	GGGCAATAAATAGTTTGTGAACAATTA	98° 10s	60° 20s	72° 3min
PaWS02746	CAAGGCGGAGGATATTTCTG	TATTTGGCTTGGGATTGAGC	98° 10s	63° 15s	72° 3min
PaWS02749	GCATATCTGAATTCACTTTGCTTC	AAGACAACTTTATTTGATTTGATGGA	98° 10s	60° 20s	72° 3min
PaZIP	CTATGGTTCGGGCGTCTAA	CAGCACAGGGAGTTCAGGTA	98° 10s	63° 30s	72° 3min

Table S2 Estimates of population differentiation between all populations and between the more densely sampled populations SE-61, SE-64 and FI-67.

Gene	Global F_{ST} for all populations	Global F_{ST} between SE-61, SE-64 and FI-67
PaAP2L3	0.01305	0.01035
PaCDF1	0.01958	0.01092
PaCOL1	0.02505	0.02748
PaMFT1	0.01426	-0.03319
PaFTL1	0.02948	0.18542
PaCCA1	-0.00537	-0.01198
PaPRR7	-0.0004	0.03142
PaPRR1	-0.01195	-0.00449
PaWS02746	-0.02664	-0.01770
PaWS02749	-0.02198	-0.00697
PaZIP	0.04879	0.03737
