# Supplementary material for: Distribution of Long-Range Linkage Disequilibrium and Tajima’s D Values in Scandinavian Populations of Norway Spruce (Picea abies)
Source: G3 (Bethesda). 2013 May 1;3(5):795–806. doi: 10.1534/g3.112.005462 (PMC3656727; doi:10.1534/g3.112.005462)
Supplement: Supporting Information [file supp_g3.112.005462_FigureS1.pdf]

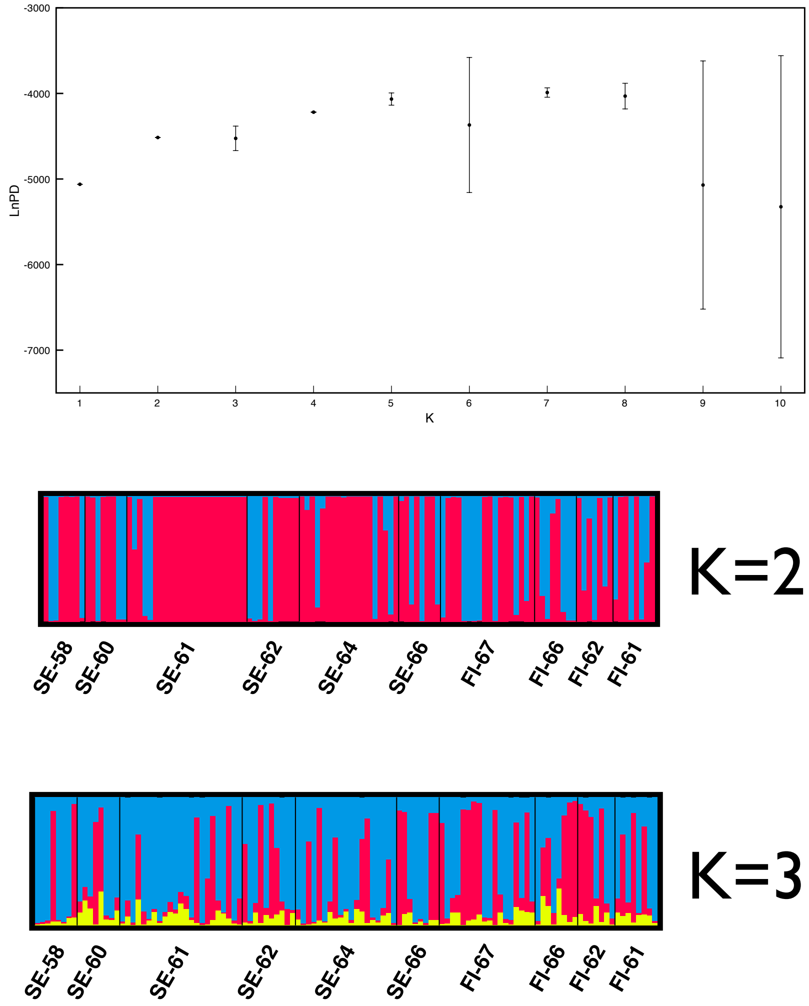

Figure S1 Top figure shows the likelihoods of estimated number of clusters (K) obtained with the program STRUCTURE. The dot represents the mean likelihood over 10 structure runs and the lines represent the standard deviation of the estimate. The bottom two figures show individual assignment results averaged over the ten runs for K=2 and K=3. Note that populations are ordered from south to north in Sweden and then north to south in Finland. The lack of geographic pattern was equally evident for all additional number of clusters tested.
