# Supplementary material for: Distribution of Long-Range Linkage Disequilibrium and Tajima’s D Values in Scandinavian Populations of Norway Spruce (Picea abies)
Source: G3 (Bethesda). 2013 May 1;3(5):795–806. doi: 10.1534/g3.112.005462 (PMC3656727; doi:10.1534/g3.112.005462)
Supplement: Supporting Information [file supp_g3.112.005462_FigureS2.pdf]

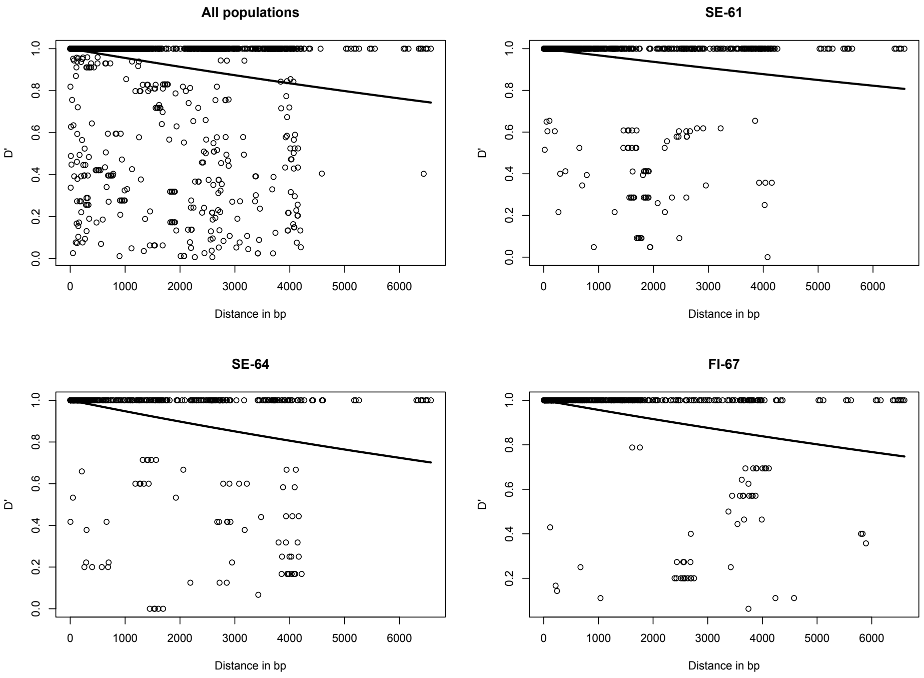

Figure S2 Plot of D´ vs. distance in base pairs across eleven loci for different subsets of populations. From top left to right bottom; a) all ten populations n=97, b) SE-61 n=20, c) SE-64 n=16 and d) FI-67 n=16.
