# Supplementary material for: Distribution of Long-Range Linkage Disequilibrium and Tajima’s D Values in Scandinavian Populations of Norway Spruce (Picea abies)
Source: G3 (Bethesda). 2013 May 1;3(5):795–806. doi: 10.1534/g3.112.005462 (PMC3656727; doi:10.1534/g3.112.005462)
Supplement: Supporting Information [file supp_g3.112.005462_FigureS3.pdf]

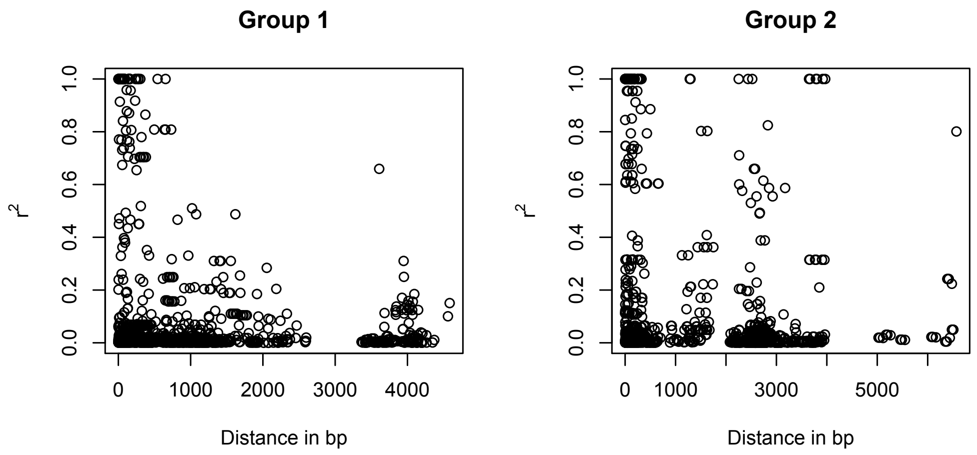

Figure S3 Plot of the squared correlation of allele frequencies (r^2) vs. distance in base pairs. Group 1 consists of the genes *PaCCA1*, *PaCDF1*, *PaCOL1* and *PaWS02746.* Group 2 consists of the genes *PaMFT1, PaFTL1, PaPRR1, PaPRR7, PaWS02749* and *PaZIP*.
