# Supplementary material for: Distribution of Long-Range Linkage Disequilibrium and Tajima’s D Values in Scandinavian Populations of Norway Spruce (Picea abies)
Source: G3 (Bethesda). 2013 May 1;3(5):795–806. doi: 10.1534/g3.112.005462 (PMC3656727; doi:10.1534/g3.112.005462)
Supplement: Supporting Information [file supp_g3.112.005462_FigureS4.pdf]

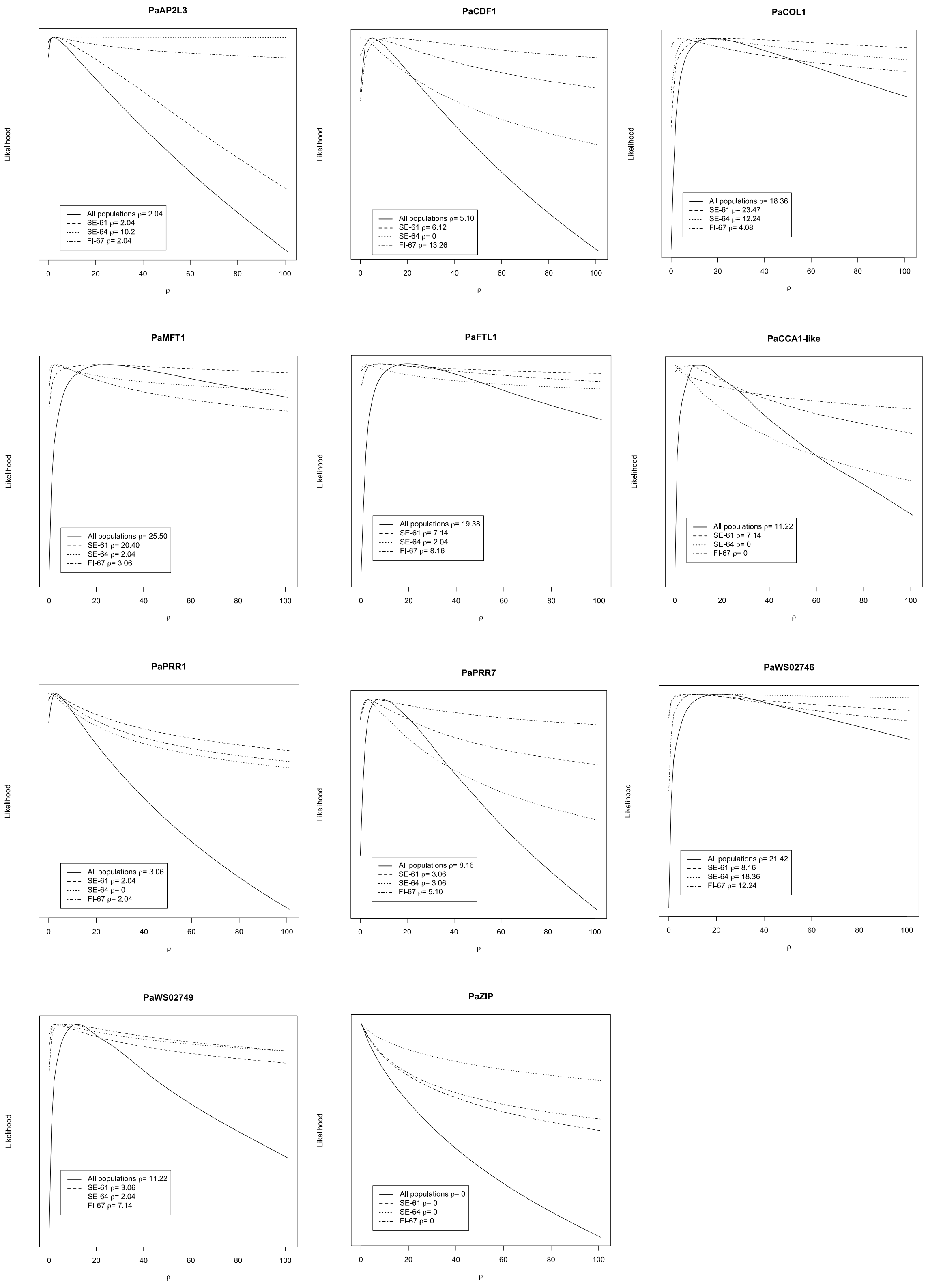

Figure S4 Per locus likelihood curves for the estimate of ρ using all populations, SE-61, SE-64 and FI-67 respectively (see legend). Curves have been rescaled for comparison.
