# Supplementary material for: Distribution of Long-Range Linkage Disequilibrium and Tajima’s D Values in Scandinavian Populations of Norway Spruce (Picea abies)
Source: G3 (Bethesda). 2013 May 1;3(5):795–806. doi: 10.1534/g3.112.005462 (PMC3656727; doi:10.1534/g3.112.005462)
Supplement: Supporting Information [file supp_g3.112.005462_TableS1.pdf]

Table S1 Table of primer sequences and PCR conditions for the genes amplified in this study. Multiple primer sequences are given in cases where the gene was amplified in overlapping fragments. All PCRs were run for 35 cycles with an initial heating at 98° for 30 seconds and ending with a 5 minute extension at 72°.

Gene	Forward primer sequence	Reverse primer sequence	Denat.	Ann.	Elong.
PaAP2L3	GGAAACAGGTTTATCTGGGTG	AAGTGACCAAAAGAAAGGCA	98° 10s	60° 20s	72° 3min
PaCDF1	TGTAGAACGGGGTGAGTTG	CTGAACCCTGCTCTTGTAATC	98° 10s	60° 20s	72° 3min
PaCOL1	CAGCAGTGGAGAATGGTGAA	CTGCATCCACATCCAATGAC	98° 10s	60° 30s	72° 30s
	CCACCCTGGTCGACTTTTTA	GGAACAACTCCGTATCCCTGA	98° 10s	60° 30s	72° 3min
PaFT1	CAAGTTCAAATTCAAAGGTAG	GGAGCATCTGGGTCTGTCAT	98° 10s	61° 30s	72° 40s
	AATGTTCGCACCTGGTTTTT	GAAGTGCTCCACAACCAACC	98° 10s	60° 30s	72° 30s
PaFT2	TGAGGACCTTCGCAACTTTT	TGTCTGATTCATTCATGGCTTC	98° 10s	63° 15s	72° 3min
PaCCA1	TATTCTCACTCTCAGCGGGG	GGACAAAACCCACTCCAGACT	98° 10s	63° 15s	72° 3min
PaPRR7	TATAAGGTTAATGAAGGGCTAG	ATAAGATGTGAATGAGAATGAA	98° 10s	59° 30s	72° 1min
	TCTTTTGGGGTAAACAACTCCT	AGAGATACACTGATAGCCTTAC	98° 10s	60° 20s	72° 3min
	AAACCTGTTAGAAAGAACGAGC	AGAGGCAAATTGTAAATATCCC	98° 10s	60° 20s	72° 3min
PaPRR1	GGCCAGTCATCCTGAGTGCGAGTCAC	GGGCAATAAATAGTTTGTGAACAATTA	98° 10s	60° 20s	72° 3min
PaWS02746	CAAGGCGGAGGATATTTCTG	TATTTGGCTTGGGATTGAGC	98° 10s	63° 15s	72° 3min
PaWS02749	GCATATCTGAATTCACTTTGCTTC	AAGACAACTTTATTTGATTTGATGGA	98° 10s	60° 20s	72° 3min
PaZIP	CTATGGTTCGGGCGTCTAA	CAGCACAGGGAGTTCAGGTA	98° 10s	63° 30s	72° 3min
