# Supplementary material for: Distribution of Long-Range Linkage Disequilibrium and Tajima’s D Values in Scandinavian Populations of Norway Spruce (Picea abies)
Source: G3 (Bethesda). 2013 May 1;3(5):795–806. doi: 10.1534/g3.112.005462 (PMC3656727; doi:10.1534/g3.112.005462)
Supplement: Supporting Information [file supp_g3.112.005462_TableS2.pdf]

Table S2 Estimates of population differentiation between all populations and between the more densely sampled populations SE-61, SE-64 and FI-67.

Gene	Global F_{ST} for all populations	Global F_{ST} between SE-61, SE-64 and FI-67
PaAP2L3	0.01305	0.01035
PaCDF1	0.01958	0.01092
PaCOL1	0.02505	0.02748
PaMFT1	0.01426	-0.03319
PaFTL1	0.02948	0.18542
PaCCA1	-0.00537	-0.01198
PaPRR7	-0.0004	0.03142
PaPRR1	-0.01195	-0.00449
PaWS02746	-0.02664	-0.01770
PaWS02749	-0.02198	-0.00697
PaZIP	0.04879	0.03737
